# Past and future effects of climate on the metapopulation dynamics of a Northeast Atlantic seabird across two centuries

**DOI:** 10.1111/ele.14479

**Published:** 2024-12-31

**Authors:** Jana W. E. Jeglinski, Holly I. Niven, Sarah Wanless, Robert T. Barrett, Mike P. Harris, Jochen Dierschke, Jason Matthiopoulos

**Affiliations:** ^1^ School of Biodiversity, One Health and Veterinary Medicine University of Glasgow Glasgow UK; ^2^ Department of Ecoscience University of Aarhus Roskilde Denmark; ^3^ UK Centre for Ecology and Hydrology Bush Estate Penicuik UK; ^4^ Department of Natural Sciences Tromsø University Museum Tromsø Norway; ^5^ Institut fuer Vogelforschung “Vogelwarte Helgoland” Helgoland Germany

**Keywords:** Bayesian state‐space models, climate change, climate forecasts, density dependence, mechanistic metapopulation models, *Morus bassanus*, near‐surface air temperature, Northern gannet, regime shifts in population regulation, sensitivity to climate change

## Abstract

Forecasting population responses to rapidly changing marine ecosystems requires mechanistic models integrating complex demographic processes, fitted to long time series, across large spatial scales. We used a Bayesian metapopulation model fit to colony census data and climatic covariates spanning 1900–2100 for all Northeast Atlantic colonies of an exemplar seabird, the Northern gannet (*Morus bassanus*) to investigate metapopulation dynamics under two climate scenarios. Fecundity varied non‐linearly with near‐surface air temperature and recruitment was depressed by sea surface temperature. We predict regime changes in density dependence as marine carrying capacities become constrained with increasing SST. Sensitivity to climate change varied across space and time, disadvantaging southwestern colonies whilst benefitting northern ones. Such sensitivity is noteworthy for a species previously assumed robust to climate change. We provide a spatial overview of climate sensitivities across a metapopulation to help with evidence‐based conservation management and open the way for similar mechanistic explorations for other colonial species.

## Graphical abstract

The authors aimed to forecast complex metapopulation responses to climate change of an exemplar seabird species, the Northern gannet (*Morus bassanus*). By using a Bayesian metapopulation model fit to very long time series of colony census data and climatic covariates (1900–2100) across very large spatial scales (all Northeast Atlantic gannet colonies), the authors found e.g. a non‐linear response of fecundity to near‐surface air temperature nsAT (a) and forecast different metapopulation trajectories for moderate and severe climate change scenarios (b). Their approach highlighted spatio‐temporal variation in sensitivity to climate change (c) that can help with evidence‐based conservation management, opening the way for similar mechanistic explorations for other colonial species.

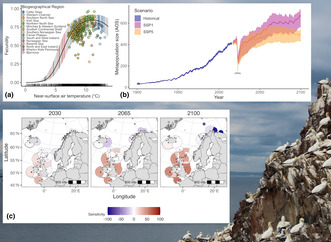

## INTRODUCTION

Anthropogenic climate change is rapidly altering the physical, biochemical and meteorological conditions of terrestrial and marine ecosystems causing direct and indirect effects on ecological communities (Doney et al., 2012; Nolan et al., 2018). Climate change scenario predictions forecast severe consequences for the future of biodiversity (Bellard et al., 2012), and already at present climate change impacts are quantifiable for large numbers of species (Pacifici et al., 2017; Poloczanska et al., 2013). Many plant and animal species show shifts in phenology (Visser & Both, 2005), whilst phenologically less sensitive groups such as seabirds respond to climate variability with detectable variation in demographic rates (Keogan et al., 2018; Oro, 2014).

Despite a rapidly increasing research focus, our understanding of species responses to climate change is far from complete and difficult to generalize. Attempts at generalizing trait‐based species responses (Pacifici et al., 2017) appear in tandem with evidence for individualistic dynamics even within broad taxonomic groups (Palmer et al., 2017; Paniw et al., 2021), suggesting the need for detailed species‐specific investigations before attempting generalization. Further, because relationships between species and their environment can vary (Southwell et al., 2021), long time series are required to detect such non‐stationary dynamics. For example, British butterfly species and seabird species off California responded differently to climate change comparing two different decadal time periods or intermediate (1–8 years) and long (8–12 years) time scales, respectively (Hyrenbach & Veit, 2003; Mair et al., 2012). For seabirds, the likelihood of detecting climate effects increased with time series length, albeit with high inter‐specific variation (Orgeret et al., 2022). Furthermore, demographic responses to climate change can be highly complex, particularly in top predators that accumulate the effects of modifications in the food webs they depend on (Frederiksen et al., 2013; Régnier et al., 2024; Trivelpiece et al., 2011). Although it is sometimes possible to detect linear relationships (Searle et al., 2022), such formulations might be inappropriate since these species track changes in the distribution and abundance of ectothermic prey with narrow thermal niches and optimal performance at intermediate conditions. For example, puffin (*Fratercula arctica*) productivity was strongly correlated with local sea surface temperature (SST), but the sign of the correlation changed three times during the 128‐year study period (Hansen et al., 2021), highlighting not just the non‐linearity of the demographic response, but also the requirement of a very long time series to detect it.

Finally, many studies cover a small geographic context compared to the species distribution. Yet, already at regional scales, fluctuations, opposing trends and variation of key drivers between different populations of the same species become evident, because climate conditions and species responses to these can vary across small spatial scales (Pardikes et al., 2015; Pincebourde et al., 2016). Furthermore, for species where local populations are connected through immigration and emigration, a metapopulation angle is required. Evidence for a metapopulation structure in seabirds is increasing (Inchausti & Weimerskirch, 2002; Jeglinski et al., 2023). Connectivity between colonies implies that impacts, regardless of their nature, may be absorbed or propagated by other parts of the colony network. Thus, forecasts of climate impacts on seabirds can be misleading if dispersal is not considered (Russell et al., 2015; Tavecchia et al., 2016).

Climate change is but one of many stressors that impact ecological communities and species groups (Caro et al., 2022) and the effects of climate change can be aggravated by increasingly intense, often localized stressors. As first step, we require a deep mechanistic understanding of the impact of climate change on populations to generate a realistic baseline for the assessment of additional anthropogenic impacts, and for robust forecasts of population dynamics that can inform conservation management.

Here, we aim to overcome obstacles in understanding climate impacts on seabirds by considering very long data time series, over large spatial scales, accounting for connectivity and for non‐linearity in demographic rates for an exemplar seabird species, the Northern gannet (*Morus bassanus*). We base our analyses on a mechanistic Bayesian state‐space model fitted to a 116‐year time series of gannet colony count data for the entire Northeast Atlantic metapopulation (Jeglinski et al., 2023). Gannets are long‐lived seabirds, characterized by high adult survival, delayed age of first breeding, single egg clutches, long nestling period and high breeding site fidelity to the often centuries‐old high‐density colonies (Nelson, 2002) and are separated into a Northwest Atlantic (6 colonies) and a Northeast Atlantic metapopulation (46 colonies in 2016) (Jeglinski et al., 2023). The gannet has been assessed as relatively insensitive to indirect climate effects and thus of limited vulnerability to climate change (Burthe et al., 2014; Furness & Tasker, 2000). Studies have described a weak positive relationship between fecundity and decreasing sea surface salinity (SLM) (Searle et al., 2022), and a negative effect of warming SST on colony growth (d'Entremont et al., 2021) but also predicted loss of breeding habitat under future climate change (Russell et al., 2015). However, the impact of climate on gannet demography has only ever been explored at small spatial scales, for short periods of time; without considering non‐linear effects or connectivity. Because of the unclear signals, expanding the spatial scale and explicitly considering more complex responses to climatic variation have been stated as priorities for future research (Searle et al., 2022).

## MATERIALS AND METHODS

### Metapopulation model

Our mechanistic Bayesian state‐space model was fit to a 116‐year time series (1900–2016) of colony census data (numbers of ‘Apparently Occupied Sites’ AOS, broadly equivalent to the number of breeding pairs) for all 53 gannet colonies in the Northeast Atlantic metapopulation, organized in 15 biogeographic regions (Jeglinski et al., 2023, Figure 1). The process component tracks the state variables $P_{n,t}$ (sizes of all colonies in the metapopulation) as they change due to demography (survival, fecundity, density‐dependent recruitment, environmental stochasticity and pre‐breeder movement driven by attraction to conspecifics). In the original model, density dependence in recruitment was either driven by terrestrial (local) constraints, or by marine (regional) limitations modelled as the effects of broad biogeographical regions.

**FIGURE 1:**
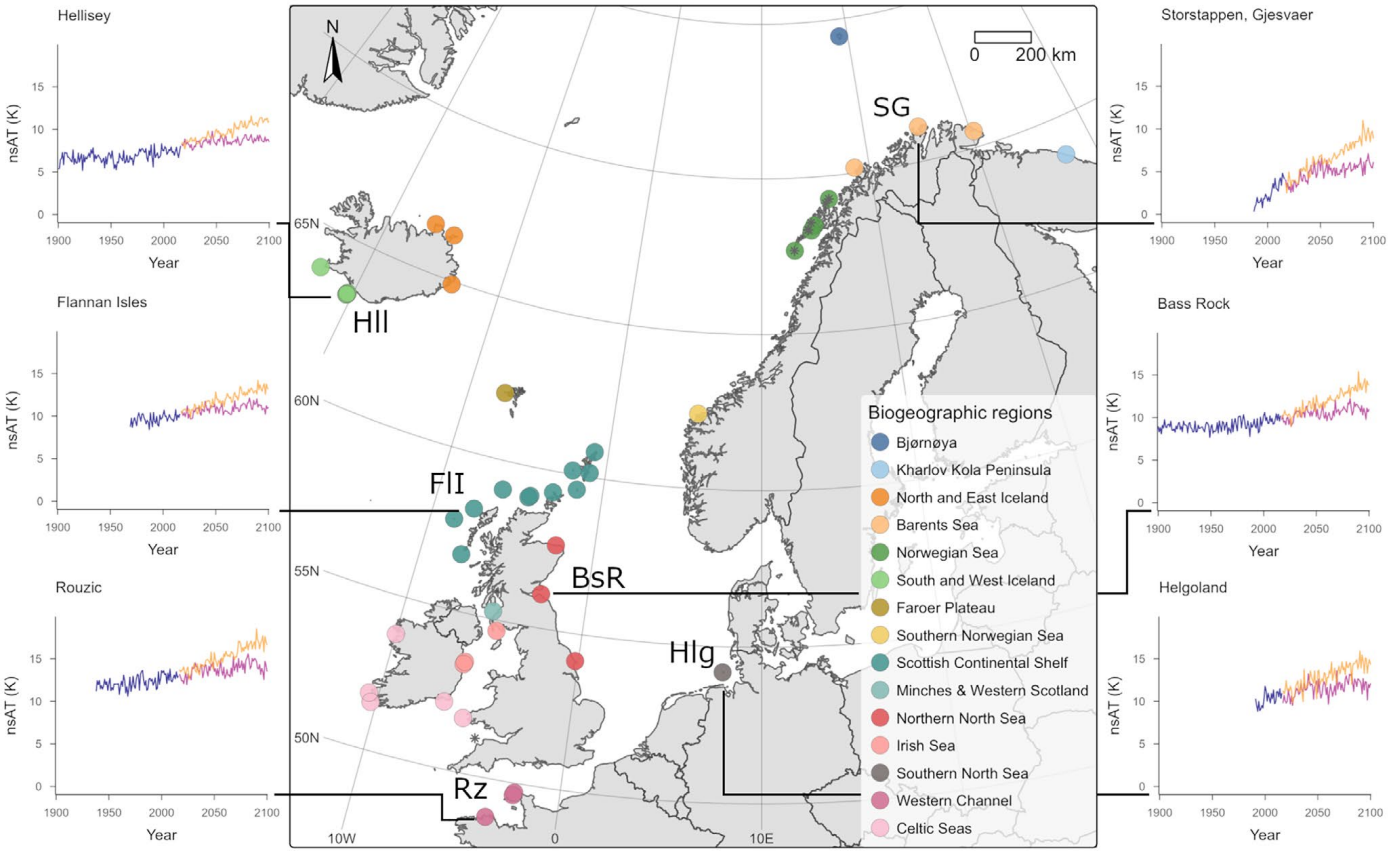
Map of the spatial distribution of the NorthEast Atlantic gannet metapopulation. Colony locations are indicated with dots coloured by biogeographic regions as in Jeglinski et al. (2023), extinct colonies are indicated with a black asterisk. Inset graphs illustrate historical (violet) and future (under SSP1 orange; under SSP5 pink) time series of near‐surface air temperature (nsAT measured in°C for six representative colonies labelled on the map.

The objective of our present study was to model the influence of past and future climate on gannet demography and long‐term dynamics for the entire metapopulation. Since the model pertained to colony dynamics during the breeding season, we tested different attributes of climate relevant for the breeding area and season as candidate covariates influencing the most important demographic processes associated with the breeding period: fecundity and density‐dependent recruitment. For a complete mathematical description of the model see Jeglinski et al. (2023). Below, we outline our key extensions to this framework.

### Climate influencing fecundity

First, we integrated into the metapopulation model all available fecundity data (345 annual means from 15 colonies, intermittently spanning 1961–2018, UK seabird monitoring program run by the JNCC, accessed at http://archive.jncc.gov.uk/smp). We modelled fecundity, that is the observed presence of a chick per nest, using a binomial distribution of the true underlying fecundity per nest, for the colonies and years with data.

Within the state‐space model, we formulated fecundity (*b*_*n*,*t*_) for each colony *n* and each year *t* as a linear and quadratic function of environmental covariates

(1) $$\;\text{logit}\left(b_{n,t}\right)=\;\alpha_{0}+\sum_{i=1}^{I}\sum_{r=1}^{2}\alpha_{\mathrm{ir}}X_{i}^{r}+\varepsilon_{t}$$

where $\alpha_{0}$ is the intercept, $X_{i}^{r}$ are the *i*‐th climate covariates raised to the power *r* (where *r* can only go up to 2), *I* is the number of covariates, $\alpha_{\mathrm{ir}}$ are the associated coefficients and $\varepsilon_{t}$ is a term for annual stochastic variation.

### Climate influencing density‐dependent recruitment

We incorporated the influence of climate on the recruitment rate by replacing the region‐specific density‐dependent parameter $\eta_{k_{n}}$ of the Jeglinski et al. (2023) model with a colony‐specific marine density dependence variable $\eta_{n}$, calculated as a function of climate covariates as in Equation (1)

(2) $$\;\text{logit}\left(\eta_{n,t}\right)=\;\beta_{0}+\sum_{i=1}^{I}\sum_{r=1}^{2}\beta_{\mathrm{ir}}X_{i}^{r}$$

As in Jeglinski et al. (2023), we used expert‐elicited information to inform the priors for the terrestrial carrying capacities *K* (i.e. qualitative estimates of the maximum number of AOS based on suitable breeding habitat available) for each colony *n* and estimated the terrestrial density‐dependent parameter $\nu_{n}$ for each colony *n*

(3) $$\nu_{n}=\frac{1}{K_{n}}\left(\alpha -\ln\left(\frac{r_{e}}{1-r_{e}}\right)\right)$$

where $r_{e}$ is the baseline recruitment rate at equilibrium (Jeglinski et al., 2023). The recruitment rate *r*_*n*,*t*_ for each colony *n* and each year *t* was either dampened by marine density dependence $\eta_{n}$ or by local (terrestrial) density dependence $\nu_{n}$

(4) $$r_{n,t}={\text{logit}}^{-1}\left(\alpha -\max\left(\nu_{n},\eta_{n,t}\right)P_{n,t}\right)$$

where α was a constant, here set to 100, to ensure that recruitment is practically 1 when both $\nu_{n}P_{n,t}$ and $\eta_{n}P_{n,t}$ are zero, that is when no density‐dependent effects applied (Jeglinski et al., 2023).

Using the marine density dependence variable calculated in Equation (2), we obtained posterior estimates of the marine carrying capacities $C_{n,t}$ for each colony *n* and each year *t*

(5) $$C_{n,t}=\frac{1}{\eta_{n,t}}\left(\alpha -\ln\left(\frac{r_{e}}{1-r_{e}}\right)\right)$$

The numerical values of $K_{n}$ and $C_{n,t}$ were estimated in AOS and thus interpretable as carrying capacities. By estimating $C_{n}$ for each time step *t*, we modelled variation in marine density dependence and carrying capacity over time, an improvement over Jeglinski et al. (2023).

By replacing the classification of colonies into biogeographic regions with the effects on demography of spatially and temporally continuous covariate data we maintained the similarity between neighbouring colonies, whilst deriving clearer insights into the drivers of similarity and gradual changes thus overall improving the realism of the model.

### Impact of high pathogenicity avian influenza HPAI

In 2022, the global gannet population was severely impacted by an outbreak of high pathogenicity avian influenza HPAI (Lane et al., 2023). Gannet survival in 2022 has only been quantified on the Bass Rock where it decreased to 0.455 (Lane et al., 2023). To produce realistic climate forecasts, we therefore applied the reduced adult survival rate in 2022 to all known affected colonies. For the only unaffected colony (Bjørnøya) we kept the regular survival term. For colonies of unknown status, we informed adult survival in 2022 with a uniform prior ranging between 0.455 and the maximum adult survival 0.94 from Jeglinski et al. (2023).

### Covariate selection and processing

We selected climate covariates based on (1) their importance in the literature, (2) historical data availability for the very long time series of our data and (3) availability for future forecasts. We considered the covariates SST, SLM, near‐surface air temperature (nsAT), near‐surface wind speed (nsWS) and precipitation (prec).

We sourced our climate data from the latest version of the Coupled Model Intercomparison Project Phase 6 (CMIP6, Table S1) (C3S & CDS, 2021; Eyring et al., 2016) based on the HadGEM3.1‐MM model (Ridley et al., 2019). This model has been found to generally reproduce many climate features in comparison to observational data over the period 1850–2014 (Andrews et al., 2020) and to outperform predecessors HadGEM2‐ES and ‐CC from the Coupled Model Intercomparison Project Phase 5 (CMIP5) in a circulation‐based performance analysis (Brands, 2021).

Covariate data outputs were available as hindcasts from 1850 to 2014 and as forecasts from 2015 to 2100, at spatial resolutions of 0.25° (SST, SLM) and ~60 km (0.83° × 0.56°, nsAT, nsWS, prec). For forecasts, we used projected climate covariates derived from two climate change scenarios: *shared socioeconomic pathway 1—2.6* (SSP1), a low‐emissions future with a >68% likelihood of limiting warming to 2°C compared to pre‐industrial conditions (Lee & Romero, 2023; UNFCCC, 2015) and *shared socioeconomic pathway 5—8.5* (SSP5), a very high emissions scenario with a >50% likelihood of warming exceeding 4°C.

As a first approximation, we used annual means of monthly covariate data for February–September, the colony attendance period of breeding gannets (Nelson, 2002). To fit our models to the full colony census time series (1900–2016), we calculated mean covariate data for 2015 and 2016 from both scenarios. We extracted covariate data using colony‐specific null utilization distribution surfaces, hereafter NUDs (described in detail in Niven et al. in preparation). NUDs, essentially at‐sea density distributions equivalent to colony home ranges in units of AOS, derive from a custom‐built mechanistic space‐use algorithm for colonial animals parameterised for the gannet that considers avoidance of land (Furness et al., 2018), within‐colony density dependence (Lewis et al., 2001) and competitive exclusion between neighbouring colonies (Wakefield et al., 2013), using reconstructed median colony sizes from Jeglinski et al. (2023) to inform time variable within‐ and between‐colony density‐dependent effects.

We produced NUDs at a 5 km resolution at 20‐year time intervals (1900, 1920, 1940, 1960, 1980, 2000, 2016), and extracted covariate data at the centroid location of each NUD grid cell, weighed covariate values by the density value of each grid cell and summed the weighted values. We utilized the same NUD surface for 20‐year time intervals. For forecasting, we used the last available NUDs (2016). Our approach balanced incorporating density‐dependent variation in colony‐specific foraging ranges over time with the time‐intensive computation of NUDs, however, it represents a major improvement in realism compared to the widespread use of static foraging ranges as boundaries for the extraction of colony‐specific environmental covariate data.

Our environmental covariate dataset consisted of five colony‐specific covariate values for each year (1900–2016 for model selection, and 2017–2100 for forecasting, see Figures [Link], [Link]), standardized to improve model convergence.

### Priors

We used priors from a normal distribution with a mean of 0 and a standard deviation of 1 for the coefficients of linear environmental effects. For relationships modelled as downward pointing parabolas (i.e. to capture optima), we used gamma priors with shape and rate of 1 imposing a positive sign for the coefficient of the linear term and a negative sign for the coefficient of the quadratic term.

### Model comparison, selection and forecasting

We initially constructed 34 models (Table 1), each including a different covariate in the linear predictors for either fecundity (Equation 1) recruitment (Equation 3) or both, and a null model that included only an intercept for both demographic rates. All other model components were identical to Jeglinski et al. (2023). We implemented the model in JAGS (Plummer, 2003) interfaced with R via the *runjags* package (Denwood, 2016). We consistently ran four parallel MCMC chains, each lasting 100,000 iterations with a burn‐in stage of 15,000, thinned to retain each 200th sample. For non‐converging models, we increased the burn‐in to a maximum of 215,000. Each model took approximately 8.5 days to run on a 4.17 GHz multicore computer. We assessed convergence of the four chains visually and by analysing the Gelman–Rubin diagnostic (Gelman & Rubin, 1992). We carried out model selection by calculating the deviance information criterion DIC (Spiegelhalter et al., 2002).

**TABLE 1:** Model selection table for all 47 models detailing model structure, model selection results and the covariate coefficients of the best fitting model.

Model	Covariate								DIC	ΔDIC
	Intercept	SST (1)	SLM (2)	nsAT (3)	nsWS (4)	Prec (5)	SST^2^	nsAT^2^		
m0.00	b, a1								25,061	0
m1.10	b, a1	b							24,854	207
m1.20	b, a1		b						24,684	377
m1.30	b, a1			b					24,724	337
m1.40	b, a1				b				NA	NA
m1.50	b, a1					b			25,001	60
m1.01	b, a1	a1							25,026	35
m1.02	b, a1		a1						25,060	1
m1.03	b, a1			a1					25,051	10
m1.04	b, a1				a1				NA	NA
m1.05	b, a1					a1			NA	NA
m2.11	b, a1	b, a1							24,799	262
m2.21	b, a1	a1	b						24,662	399
m2.31	b, a1	a1		b					24,603	458
m2.41	b, a1	a1			b				NA	NA
m2.51	b, a1	a1				b			24,966	95
m2.12	b, a1	b	a1						NA	NA
m2.22	b, a1		b, a1						24,676	385
m2.32	b, a1		a1	b					NA	NA
m2.42	b, a1		a1		b				NA	NA
m2.52	b, a1		a1			b			24,986	75
m2.13	b, a1	b		a1					24,837	224
m2.23	b, a1		b	a1					NA	NA
m2.33	b, a1			b, a1					24,706	355
m2.43	b, a1			a1	b				24,863	198
m2.53	b, a1			a1		b			24,968	93
m2.14	b, a1	b			a1				NA	NA
m2.24	b, a1		b		a1				NA	NA
m2.34	b, a1			b	a1				NA	NA
m2.44	b, a1				b, a1				24,845	216
m2.54	b, a1				a1	b			NA	NA
m2.15	b, a1	b				a1			24,825	236
m2.25	b, a1		b			a1			24,670	391
m2.35	b, a1			b		a1			24,975	86
m2.45	b, a1				b	a1			NA	NA
m2.55	b, a1					b, a1			24,702	359
m2.31^2	b, a1	a1		b			a1		NA	NA
m2.3^21	b, a1	a1		b				b	24,366	695
m2.3^21^2	b, a1	a1		b			a1	b	NA	NA
m3.311	b, a1	a1, b		b					NA	NA
m3.321	b, a1	a1	b	b					NA	NA
m3.341	b, a1	a1		b	b				NA	NA
m3.351	b, a1	a1		b		b			24,580	481
m3.3^211	b, a1	a1, b		b				b	24,367	694
m3.3^212	b, a1	a1	b	b				b	24,323	738
m3.3^241	b, a1	a1		b	b			b	24,299	762
**m3.3^251**	**b, a1**	**a1**		**b**		**b**		**b**	**24,282**	**779**
	Median	Lower 95		Upper 95						
Intercept b	0.969	0.126		1.73						
nsAT b	1.546	1.43		1.67						
nsAT b^2	0.848	0.757		0.938						
prec b	0.139	0.095		0.186						
Intercept a1	−6.619	−6.72		−6.54						
SST a1	0.881	0.808		0.96						

*Note*: We investigated the demographic rates fecundity (indicated by b) and recruitment (indicated by a1) and for each demographic rate linear or quadratic mechanistic relationships with five covariates: (1) near‐surface air temperature (nsAT), (2) sea surface temperature (SST), (3) sea surface salinity (SLM), (4) near‐surface wind speed (nsWS) and (5) precipitation (prec). Models are ordered according to the number and nature of contributing covariates (1–5). ΔDIC indicates the difference between each model and the null model, the best model therefore had the highest ΔDIC and is indicated in bold. NA values indicate non‐convergence.

We then compared a suite of 11 more complex, multi‐covariate models. First, for the best model (linear term for nsAT in fecundity and linear term for SST in recruitment, Table 1), we added a quadratic term to either or both linear terms (three models) to explore potential optima in the respective demographic rate. The only model that converged contained a quadratic term for nsAT in the linear predictor of fecundity. We then added a second linear covariate (SST, SLM, nsWS, prec) to the linear predictor for fecundity and compared models that had a linear term for nsAT (4 models, Table 1) and a quadratic term for nsAT (4 models, Table 1).

We used the model with the lowest DIC (Table 1) to forecast gannet metapopulation dynamics for the contrasting climate scenarios SSP1 and SSP5 from 2017 to 2100.

## RESULTS

In total, we investigated 47 models that included single or multiple covariates in either linear or non‐linear form acting on recruitment rate, fecundity or both, and a null model (Table 1). All models fitted the data better than the null model (Table 1). The model with the lowest DIC incorporated mechanistic relationships with climate for both demographic rates: nsAT and rainfall influenced fecundity and SST influenced recruitment (Table 1).

**FIGURE 2:**
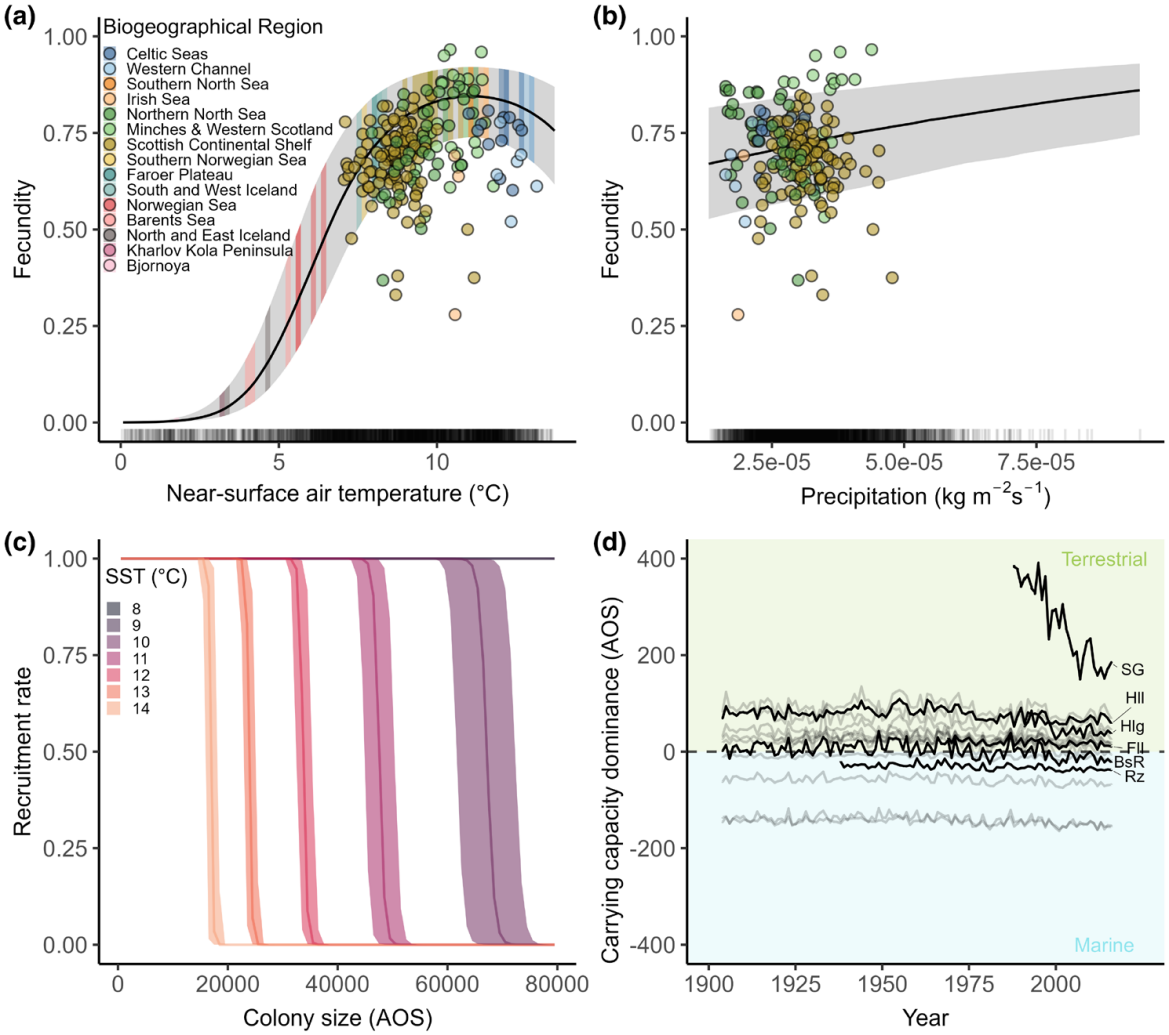
Relationship between demographic rates and environmental covariates in the best fitting model. (a) Non‐linear relationship between fecundity and near‐surface air temperature (nsAT) in°C. Dots represent all available empirical fecundity data coloured by biogeographic region (see legend) at the colony‐specific mean nsAT during the year of data collection. Mean nsAT experienced at colonies in 2016 is illustrated by coloured bands of the credible interval zones by biogeographic region (see legend) (b) linear positive relationship between fecundity and precipitation (median gradient of 0.14) and dots representing all available fecundity estimates as in (a). (c) Relationship between recruitment rate and colony size coloured by mean sea surface temperature (SST). Recruitment rate drops at lower temperatures when the population of a given colony at carrying capacity is higher. Rug plots below panels (a), (b) and (c) indicate distribution of colony‐specific environmental values 1900–2016. (d) Changes in carrying capacity dominance, defined as the difference between the marine and terrestrial posterior carrying capacity for the 15 most populated colonies in 2016, and the example colonies Helgoland (Hlg) and Storstappen, Gjesvaer (SG). Colonies under marine density‐dependent regulation are constrained by the relationship shown in (c). Background colours in green pertain to terrestrial regulation, areas in blue to marine regulation. Y‐axes values in (c) and (d) AOS/10^3^.

Fecundity varied non‐linearly with nsAT (Figure 2a). The model with nsAT as a linear effect was of considerably poorer fit (ΔDIC = 298) and three of four more complex models with a second covariate in the linear predictor only converged when nsAT was modelled as quadratic term, highlighting the importance of the non‐linear relationship between fecundity and nsAT (Table 1).

Fecundity increased with increasing nsAT but peaked at 0.84 at 11.15°C, so that the relationship between fecundity and nsAT was positive for colonies experiencing mean nsAT below and at the optimum, but negative for colonies experiencing higher temperatures (Celtic Seas, Western Channel, Figure 2a). In addition, rainfall had a small positive effect on fecundity (Figure 2b).

Recruitment is a density‐dependent process and thus generally decreased with increasing colony size. However, the recruitment rate was also influenced by SST: colonies started to experience density‐dependent limitations at smaller sizes as SST increased (Figure 2c). The influence of SST was relevant for colonies where marine density dependence dominated over terrestrial density dependence (7 out of 46 in 2016) and ultimately meant that increasing SST reduced the carrying capacities of these colonies. Furthermore, the regulating nature (terrestrial or marine) and strength of density dependence varied considerably over time (Figure 2d).

**FIGURE 3:**
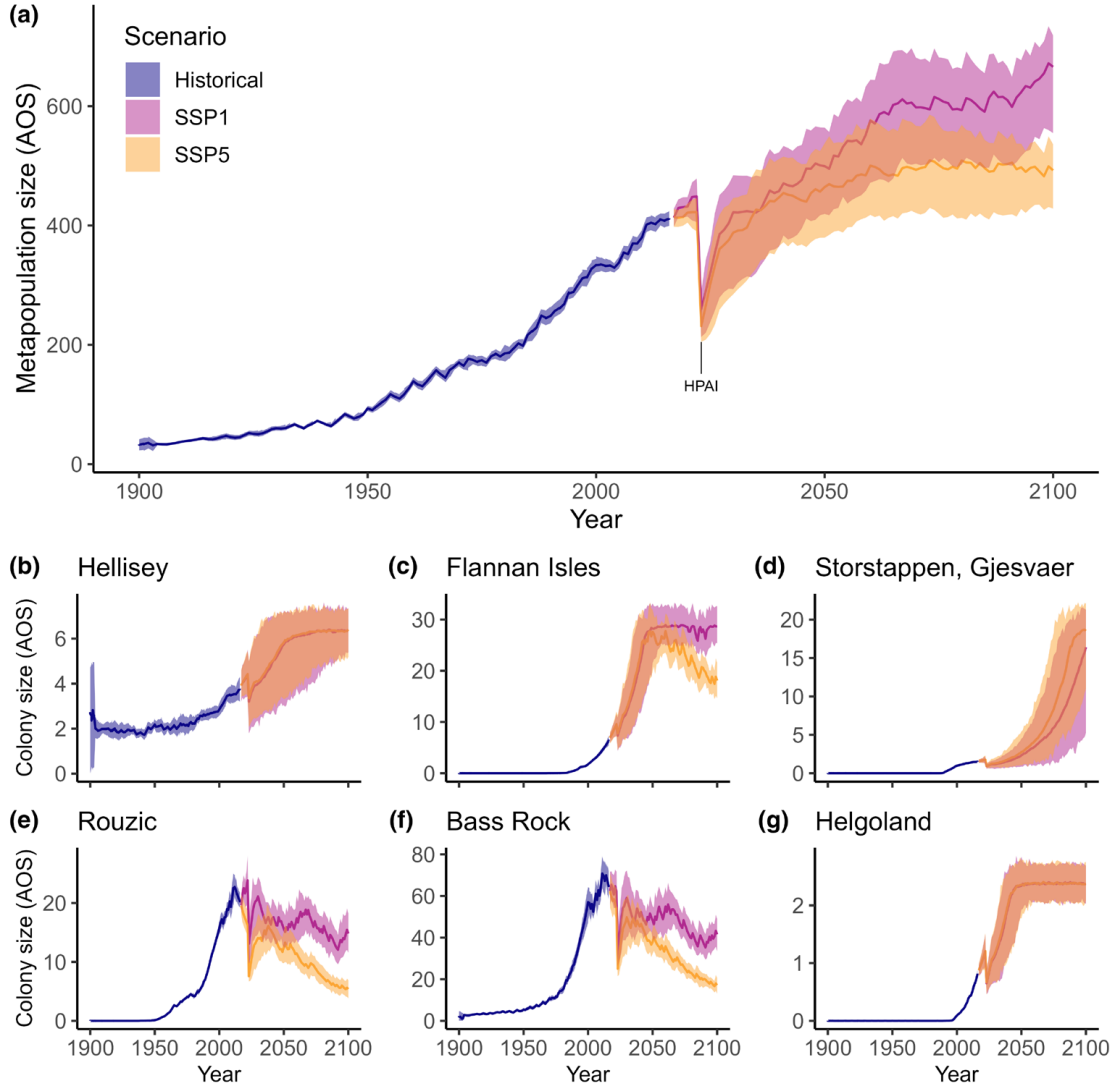
Hindcasts and forecasts for the two climate scenarios SSP1 (pink) and SSP5 (orange) for (a) the Northeast gannet metapopulation and (b–g) six exemplary colony‐specific population trajectories, highlighting four distinct patterns in future trajectories (see Results). Y‐axes values in AOS/10^3^.

Forecasting metapopulation growth into the future led to different trajectories depending on climate scenario. Following a drastic decline due to the HPAI outbreak in 2022 for both scenarios, the metapopulation is projected to increase from 2030 onwards at an average rate of 0.83% pa under SSP1, while growth is predicted to plateau around 2055 under SSP5 (Figure 3a). However, this large‐scale trend obscured strongly divergent patterns at the colony level. Forecasts for 46% of colonies did not differ and plateaued at their respective carrying capacities well before 2100 (Figure 3b,g). Forecasts for 22% of colonies were initially indistinguishable but increasingly diverged as time progressed, plateauing under SSP1 and decreasing under SSP5 (Figure 3c). 17% of colonies were forecast to increase in size without levelling off, with steeper increases projected for SSP5 (Figure 3d) and 15% of colonies were forecast to decrease drastically under both climate scenarios, with less steep declines under SSP1 (Figure 3e,f, for all colony growth trajectories see Figure S6).

**FIGURE 4:**
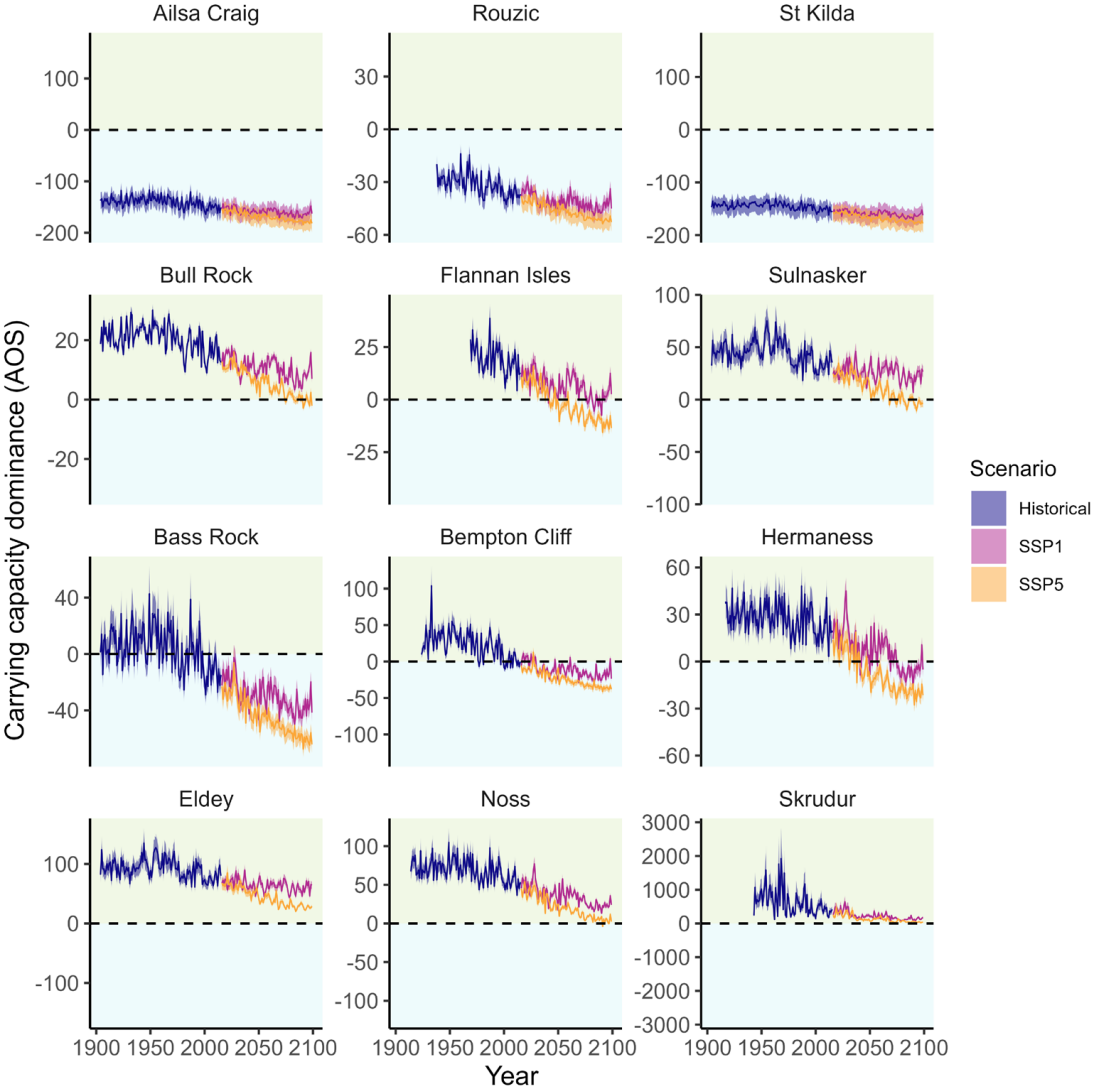
Carrying capacity dominance trajectories of 12 of the 16 most populous gannet colonies (67% of the metapopulation size) in 2016. Carrying capacity dominance (AOS/10^3^), defined as the difference between the estimated colony‐specific marine and terrestrial carrying capacities, is shown for the hindcast and the SSP1 and SSP5 scenario forecasts. To facilitate illustration, the background area (above 0) in green indicates terrestrial regulation, background area in blue (below 0) to marine regulation.

This divergence of colony‐specific responses to future climates is driven by the non‐linear response of fecundity to nsAT since increasingly, colonies will experience temperatures beyond the optimum as the climate warms (Figure 2a). In addition, rising SST increasingly influenced recruitment since the dominant form of density‐dependent regulation is predicted to change over time: for both climate scenarios, and particularly under SSP5, 20% of formerly terrestrially regulated colonies became regulated by the marine environment as marine carrying capacities decreased due to the warming climate (Figure 4). In general, marine carrying capacities were predicted to be consistently lower under SSP5 compared to SSP1, and these differences became more exacerbated over time (Figure 4; Figure S7).

**FIGURE 5:**
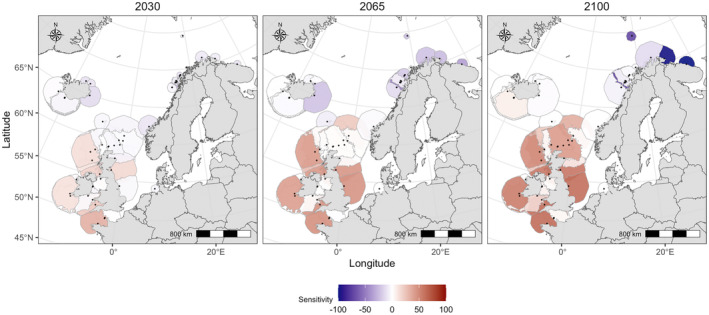
The sensitivity of all colonies (black dots) in the Northeast Atlantic metapopulation to changes in climatic conditions assessed for the short‐term (2030), intermediate (2065) and longer‐term (2100) future. Sensitivity is defined as the % difference in median posterior colony size between the SSP1 and SSP5 climate change scenario and illustrated as coloured colony‐specific NUDs with red colours indicating reductions and blue colours increases in colony size under SSP5 compared to SSP1. Time series of future colony size changes are illustrated by plotting NUDs based on posterior median colony size estimates for 2030, 2065 and 2100 under the SSP1 scenario. The colour gradient is constrained between −100% and 100% to reduce the influence of a single outlier, the Kharlov Kola Peninsula colony which is predicted to be 150% larger under SSP5 compared to SSP1 in 2100.

Because of the inferred central role of nsAT, SST and rainfall in metapopulation regulation of the gannet, the metapopulation is sensitive to climate change. Sensitivity (the per cent difference in forecast posterior colony size between SSP1 and SSP5) increased over time but varied in space (Figure 5). Already in the short‐term (2030), colonies at the southwestern extent of the metapopulation were forecast to be smaller under SSP5 than they would be under SSP1, while colonies at the northern extent were forecast to be slightly larger, benefitting from the more extreme climate change scenario. These large‐scale differences in sensitivity became more pronounced over time (2065 and 2100) with increasing sensitivity in the southern and central metapopulation and an increasing beneficial effect at the northern extent, however, overall, the bulk of the metapopulation highlighted a negative impact of climate change on the gannet (Figure 5).

## DISCUSSION

We set out to better understand the impact of climate on the demography and metapopulation dynamics of an exemplar seabird species, the gannet. Integrating a very long time series of colony census data and hindcast climatic covariates into a mechanistic metapopulation model for the entire Northeast Atlantic, we found clear evidence for climate‐driven metapopulation regulation. Our findings highlight the importance of considering very long time series (here, this allowed us to uncover changes in density‐dependent regulation), and very large spatial scales (here, this revealed opposing trends in sensitivity to climate change for colonies at different extremes of the geographic distribution of the Northeast Atlantic metapopulation).

Gannets were previously considered relatively insensitive to climate change or indeed suggested to benefit from it (Burthe et al., 2014; Searle et al., 2022); however, we show here that the scope of assessment may change this conclusion. By ‘zooming out’ beyond the narrow focus of a study region, we found a non‐linear relationship between fecundity and nsAT, that is different directions of this relationship for different climate regimes, which are expected to become exacerbated with progressive climate warming (Figures 2a and 5). By expanding the time horizon, we showed that for colonies dominated by marine density dependence, increasing SST reduced recruitment, and we predicted that marine regulation will increase as warming SST decreases marine carrying capacities of colonies (Figure 4). Instead of standing out as rare ‘winners’ or clear losers of climate change (Russell et al., 2015), gannet responses resemble a mosaic of colony‐specific sensitivities to climate change varying across space and time.

The impact of climate change on marine top predators is usually considered indirect, via changes in prey distribution and abundance (Jenouvrier, 2013; Sydeman et al., 2015). Our findings highlight the impact of warming air temperatures as an important additional stressor that is rarely explicitly considered and rarely linked with population dynamics (Mauck et al., 2018). As temperatures warm, parents may increasingly need to trade‐off offspring protection and provisioning against their own thermoregulatory needs. For example, parental nest attendance decreased with rising air temperatures, increasing breeding failure in common guillemots (*Uria aalge*) because abandoned chicks died of heat stress or predation (Olin et al., 2024). High air temperatures can also lead to trade‐offs between foraging efficiency and thermoregulation impacting fledgling condition and fledging success in terrestrial birds (van de Ven et al., 2020). On the population level, these findings not only highlight mechanistic impacts of air temperature on an important demographic rate, but also that climate change may influence the suitability of breeding habitat and thus modify its carrying capacity. For seabirds, gathering empirical evidence on the variation in thermal suitability of nest site microclimate will allow us to extend our model to also investigate non‐stationarity in terrestrial carrying capacity.

We found evidence for latitudinal differences of climate change impacts across the Northeast Atlantic metapopulation, where colonies at the Southwestern extent are predicted to decrease under a more extreme climate change scenario, while colonies in the North appear to benefit from it (Figure 5). There is already empirical support for such large‐scale differences in sensitivity: a disproportionally large foraging effort in gannets from Rouzic, the southernmost gannet colony globally (Grémillet et al., 2006), a negative impact of decreasing SST and declining mackerel availability on fecundity in Cape St. Mary, one of the southernmost colonies in the NorthWest Atlantic metapopulation (d'Entremont et al., 2021) and differences in frequency of recent colonization attempts between these regions (eight attempts in Norway since 2016, G. Systad, personal communication, three in the UK and one in Ireland, Seabird Monitoring Programme https://app.bto.org/seabirds/public/data.jsp, accessed 7th March 2024).

We note here that even though our model is spatially implicit, that is considers the effect of covariates at colony locations, it is not spatially explicit and thus cannot predict future colonisations of novel sites (due to computational constraints of a very large spatial extent). However, the proportional contribution from newly formed colonies to the gannet metapopulation size stays very small for decades and we are therefore confident that our metapopulation‐level predictions are robust to this limitation in our model structure. Our findings suggest that northern regions may act as ‘refugia from climate change’ (Ashcroft, 2010) for the gannet. Most of these colonies though appear to stay small and all recent extinctions happened in Norway (Jeglinski et al., 2023). The drivers of these dynamics are poorly understood: high predation pressure from white‐tailed eagles (*Halieetus albicilla*) has been suggested (Barrett, 2008; Barrett et al., 2017; Pettex et al., 2015), and colony exposition to high swells may play a role. Empirical research into and quantification of these drivers is required before this region can really be considered a climate change refugium for the gannet.

Our study focusses on changes in average climate conditions to better understand the general impact of large‐scale long‐term forecasts and differing climate change scenarios on the metapopulation dynamics of an exemplar seabird. Human‐induced climate change is additionally attributed to the increasing frequency of extreme climate events such as heatwaves and storms (Ummenhofer & Meehl, 2017), and, concomitantly, evidence for animal responses and associated population‐level consequences is increasing (Sergio et al., 2018). For example, marine heatwaves have been shown to cause massive seabird die‐offs (Schoen et al., 2024). However, extreme events may also have stabilizing effects on population dynamics by interacting with density dependence and age structure as demonstrated in high arctic reindeer (*Rangifer tarandus*) (Hansen et al., 2019). The potential collapse of the Atlantic Meridional Overturning Circulation AMOC, leading to large‐scale changes in oceanographic conditions with likely impacts on marine ecosystems in the Northeast Atlantic (Ditlevsen & Ditlevsen, 2023) may also be considered an extreme event, though it should be noted that IPCC considers AMOC collapse within the 21‐century unlikely (IPCC, 2023). We can explore such counterfactuals in future applications of our flexible modelling framework by contrasting climate extremes with our climate‐driven metapopulation predictions as a baseline. We expect that adding such stochastic impacts will enhance the already detectable impact of climate variability on gannet metapopulation dynamics shown here.

We note two important future extensions to our model that may modify colony‐specific responses to climate. Firstly, metapopulation connectivity in our model is driven by conspecific attraction but does not account for potential decay with distance. Support for distance‐dependence structuring connectivity in seabirds metapopulations is not unequivocal (e.g. Fernández‐Chacón et al., 2013; Genovart et al., 2013) but understanding its role in gannets will be important for future studies aiming to understand colony‐specific disturbances, associated ripple effects and source sink dynamics. Secondly, since our model incorporates breeding season dynamics only, we focus on climatic drivers of fecundity and recruitment, since immature and adult survival impacts predominantly happen in the non‐breeding season, for example climate may impact seabird survival during winter due to the inability of birds to feed during extreme weather (Clairbaux et al., 2021). Drastically decreasing return rates of instrumented gannets for the southernmost colony Rouzic in the Northeast Atlantic metapopulation (Grémillet et al., 2020) suggest that overwinter dynamics, even if not related to climate, may play an important additional role in this species. Such future extension of our model requires detailed empirical data on colony‐specific space‐use patterns during the migration and overwintering period.

### Anticipatory and spatially explicit conservation policy

Seabird metapopulations are threatened by multiple direct and indirect, global and localized stressors (Dias et al., 2019). Anticipatory and spatially explicit conservation management requires an appreciation that sensitivities and vulnerabilities may differ across space and time. Here, we quantify and illustrate variation in climate sensitivity for all colonies in the Northeast Atlantic metapopulation (Figure 5). Because of these differences, the same type of impact (e.g., windfarm construction, fisheries bycatch, discard regimes, disease outbreak) could have a detrimental or negligible effect. For our study species, conservation managers can use our sensitivity maps as guideline for prioritizing management decisions, for example aiming to reduce the influence of manageable deleterious effects in colonies that experience high sensitivity to climate. Whole‐metapopulation models such as ours that are holistically fitted to integrated data explicitly allow deep‐future predictions and can, for example, forecast colony‐specific impacts of offshore windfarm mortality, future disease outbreaks or bycatch mortality on the baseline of metapopulation responses to a changing climate. Such horizon scanning, now becoming a realistic expectation in the toolbox of environmental managers, can allow minimal mitigation in the present to have maximal benefits for species viability in the future.

### Ecological insights

We extract several important ecological conclusions from our findings. Firstly, we find further evidence for the suggestion that “moderate is better” (Sydeman et al., 2015). Animals may generally respond to climate in a non‐linear way thus ecologists should consider optimal response curves, and look to the past for evidence that such optima may change over time (Hansen et al., 2021).

Secondly, our results emphasize the risk of drawing misleading conclusions when analysing and generalizing signals from small subsets of a (meta)population. Direction, strength and relevance of responses to climate may differ widely between local populations, patches or colonies, and depend on geographic location, size, connectivity and patch saturation compared to its carrying capacity.

Thirdly, we highlight the importance of considering temporal changes which require long time series, both for past and future predictions. Such non‐stationarity, for example here shown for marine carrying capacities, is not just important for realistic population models, but paramount for conservation management because it gives us a quantitative idea of changes to the margin within which a colony or population would be able to absorb additional stressors.

### Outlook and conclusions

We built mechanistic relationships with climate and demography into an existing metapopulation model to accelerate progress in robust assessment of the multiple anthropogenic and natural threats to an exemplar seabird species. A spatially explicit understanding and quantification of these threats while accounting for realistic metapopulation projections under climate change is paramount for informed precautionary conservation management decisions, both for stressors that are somewhat controllable (e.g., windfarm placement, fisheries bycatch) and for additive and multiplicative impacts beyond direct control (e.g. emerging disease). We encourage similar mechanistic explorations for other colonial species taking heed of the ecological ‘take home messages’ formulated above.

## AUTHOR CONTRIBUTIONS

JWEJ, HIN and JM conceived the ideas, JWEJ, HIN and JM designed the methodology, in discussion with SW and MPH; SW, JWEJ, MPH, JD and RTB collected and collated the data; HIN ran the analyses with support from JWEJ and JM; JWEJ wrote the manuscript. All authors contributed critically to the drafts and gave final approval for publication.

### PEER REVIEW

The peer review history for this article is available at https://www.webofscience.com/api/gateway/wos/peer‐review/10.1111/ele.14479.

## Supporting information


**Figure S1:** Historical (violet) and future (under climate change scenario SSP1 in green, under SSP5 in yellow) time series of sea surface temperature for all extant gannet colonies of the Northeast Atlantic metapopulation.

**Figure S2:** Historical (violet) and future (under climate change scenario SSP1 in green, under SSP5 in yellow) time series of sea surface salinity for all extant gannet colonies of the Northeast Atlantic metapopulation.

**Figure S3:** Historical (violet) and future (under climate change scenario SSP1 in green, under SSP5 in yellow) time series of near‐surface air temperature for all extant gannet colonies of the Northeast Atlantic metapopulation.

**Figure S4:** Historical (violet) and future (under climate change scenario SSP1 in green, under SSP5 in yellow) time series of precipitation for all extant gannet colonies of the Northeast Atlantic metapopulation.

**Figure S5:** Historical (violet) and future (under climate change scenario SSP1 in green, under SSP5 in yellow) time series of near‐surface wind speed for all extant gannet colonies of the Northeast Atlantic metapopulation.

**Figure S6:** Posterior estimates of median colony sizes and credible interval spanning the years 1900–2016 (violet) and future colony size predictions under climate change scenario SSP1 (green) and under SSP5 (yellow) for all extant gannet colonies of the Northeast Atlantic metapopulation.

**Figure S7:** Posterior estimates of colony carrying capacities spanning the years 1900–2016 (violet) and future predictions under climate change scenario SSP1 (green) and under SSP5 (yellow) for all extant gannet colonies of the Northeast Atlantic metapopulation. Background colours in green pertain to terrestrial regulation, areas in blue to marine regulation.

**Table S1:** Model ID and institution ID of model used for covariate data.

## Data Availability

Our code and data are openly available at https://doi.org/10.5281/zenodo.10785569 and https://doi.org/10.5281/zenodo.12513978, respectively.
