# Supplementary figures and images for: Past and future effects of climate on the metapopulation dynamics of a Northeast Atlantic seabird across two centuries
Source: Ecol Lett. 2024 Dec 31;27(12):e14479. doi: 10.1111/ele.14479 (PMC11686948; doi:10.1111/ele.14479)

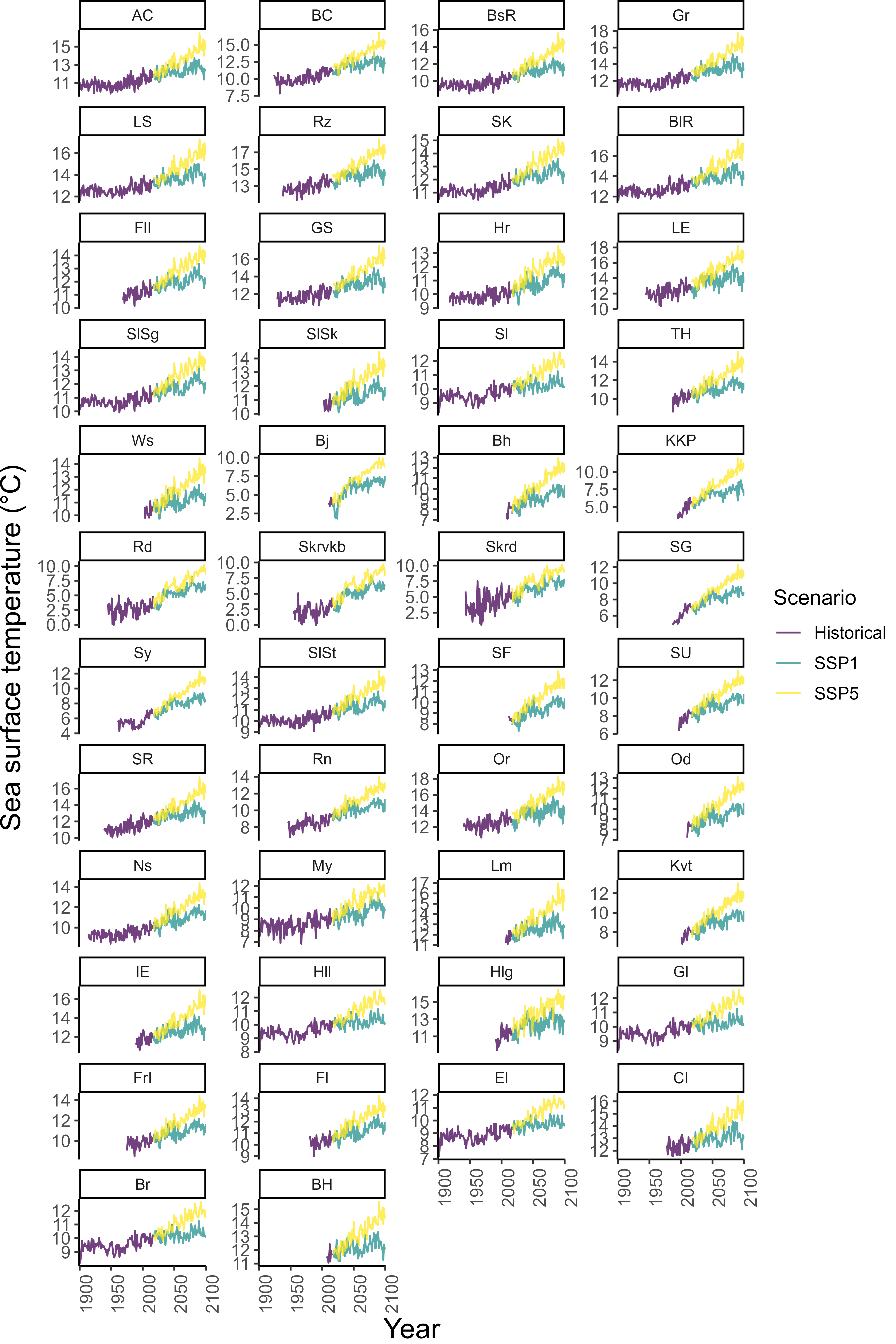

Supplement: Supplementary file 1 — Figure S1: Historical (violet) and future (under climate change scenario SSP1 in green, under SSP5 in yellow) time series of sea surface temperature for all extant gannet colonies of the Northeast Atlantic metapopulation. [file ELE-27-0-s004.tiff]

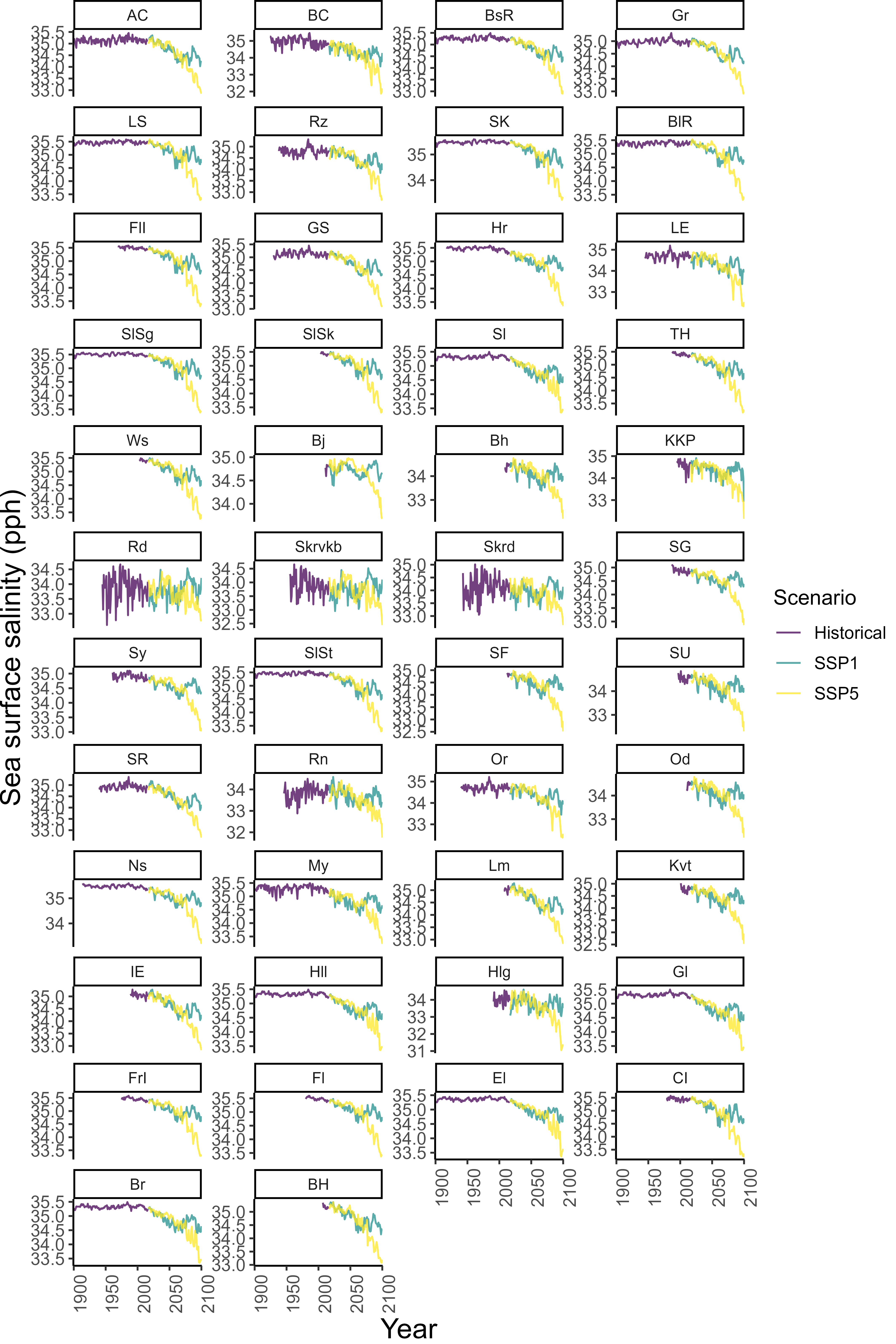

Supplement: Supplementary file 2 — Figure S2: Historical (violet) and future (under climate change scenario SSP1 in green, under SSP5 in yellow) time series of sea surface salinity for all extant gannet colonies of the Northeast Atlantic metapopulation. [file ELE-27-0-s008.tiff]

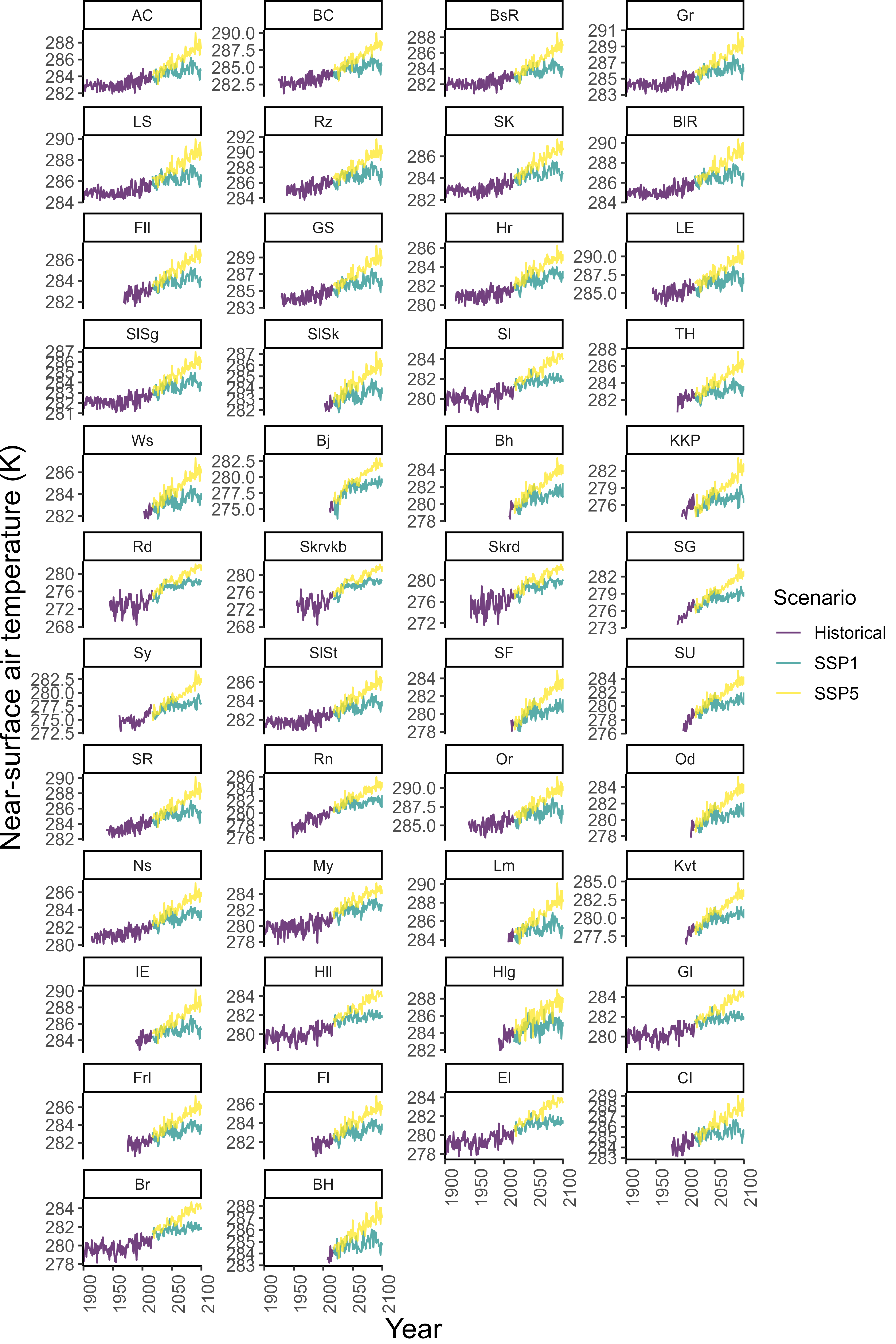

Supplement: Supplementary file 3 — Figure S3: Historical (violet) and future (under climate change scenario SSP1 in green, under SSP5 in yellow) time series of near‐surface air temperature for all extant gannet colonies of the Northeast Atlantic metapopulation. [file ELE-27-0-s001.tiff]

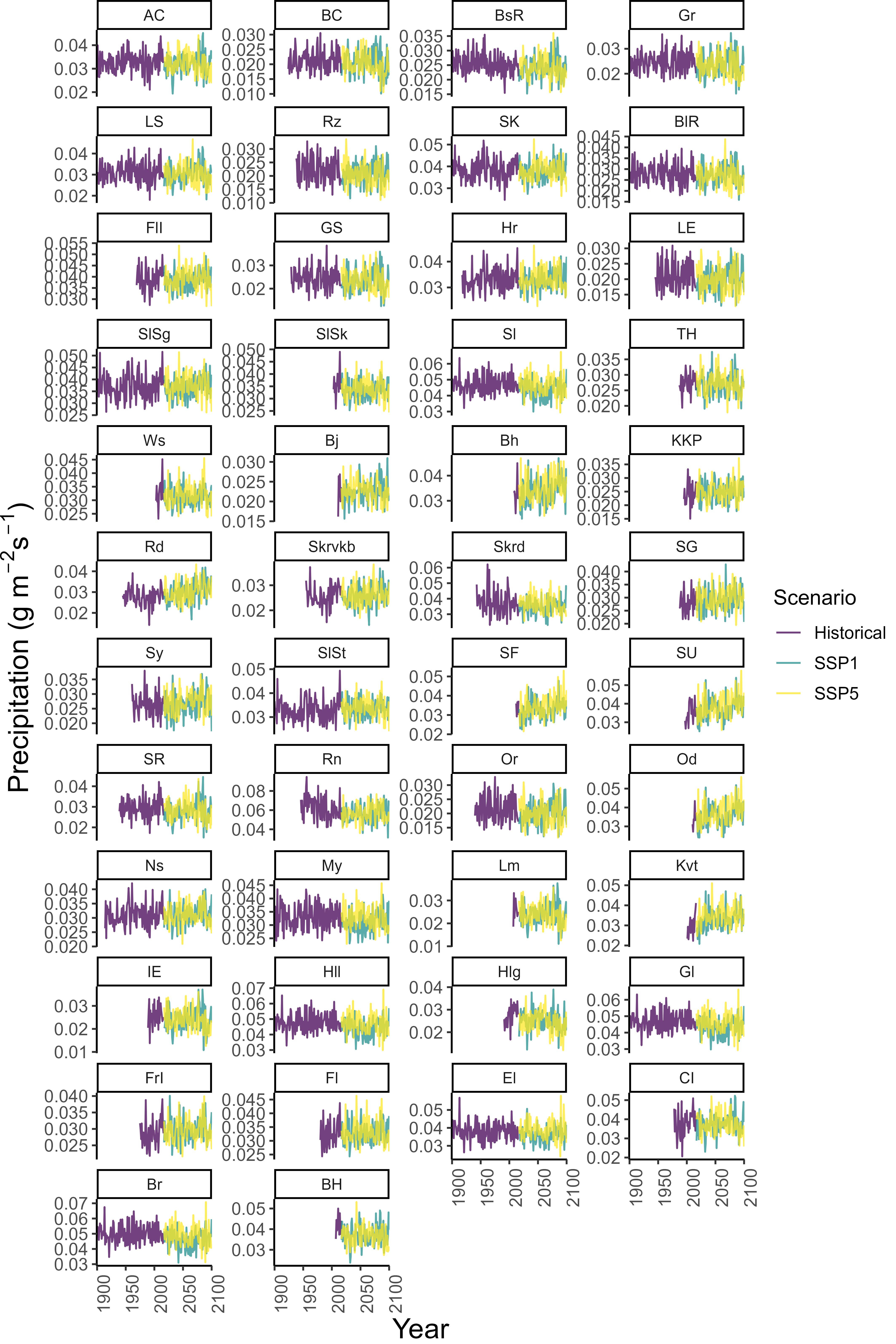

Supplement: Supplementary file 4 — Figure S4: Historical (violet) and future (under climate change scenario SSP1 in green, under SSP5 in yellow) time series of precipitation for all extant gannet colonies of the Northeast Atlantic metapopulation. [file ELE-27-0-s006.tiff]

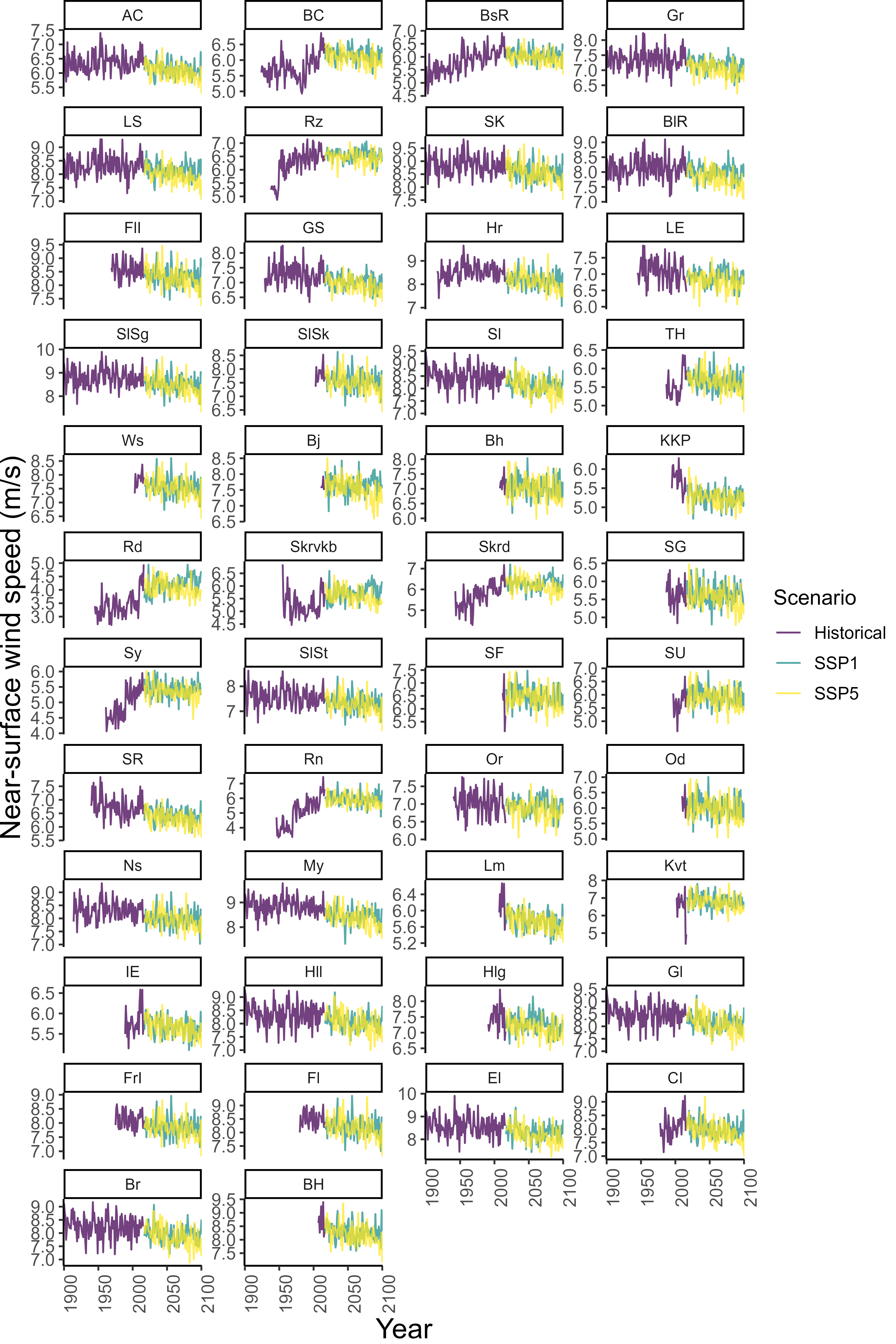

Supplement: Supplementary file 5 — Figure S5: Historical (violet) and future (under climate change scenario SSP1 in green, under SSP5 in yellow) time series of near‐surface wind speed for all extant gannet colonies of the Northeast Atlantic metapopulation. [file ELE-27-0-s005.tiff]

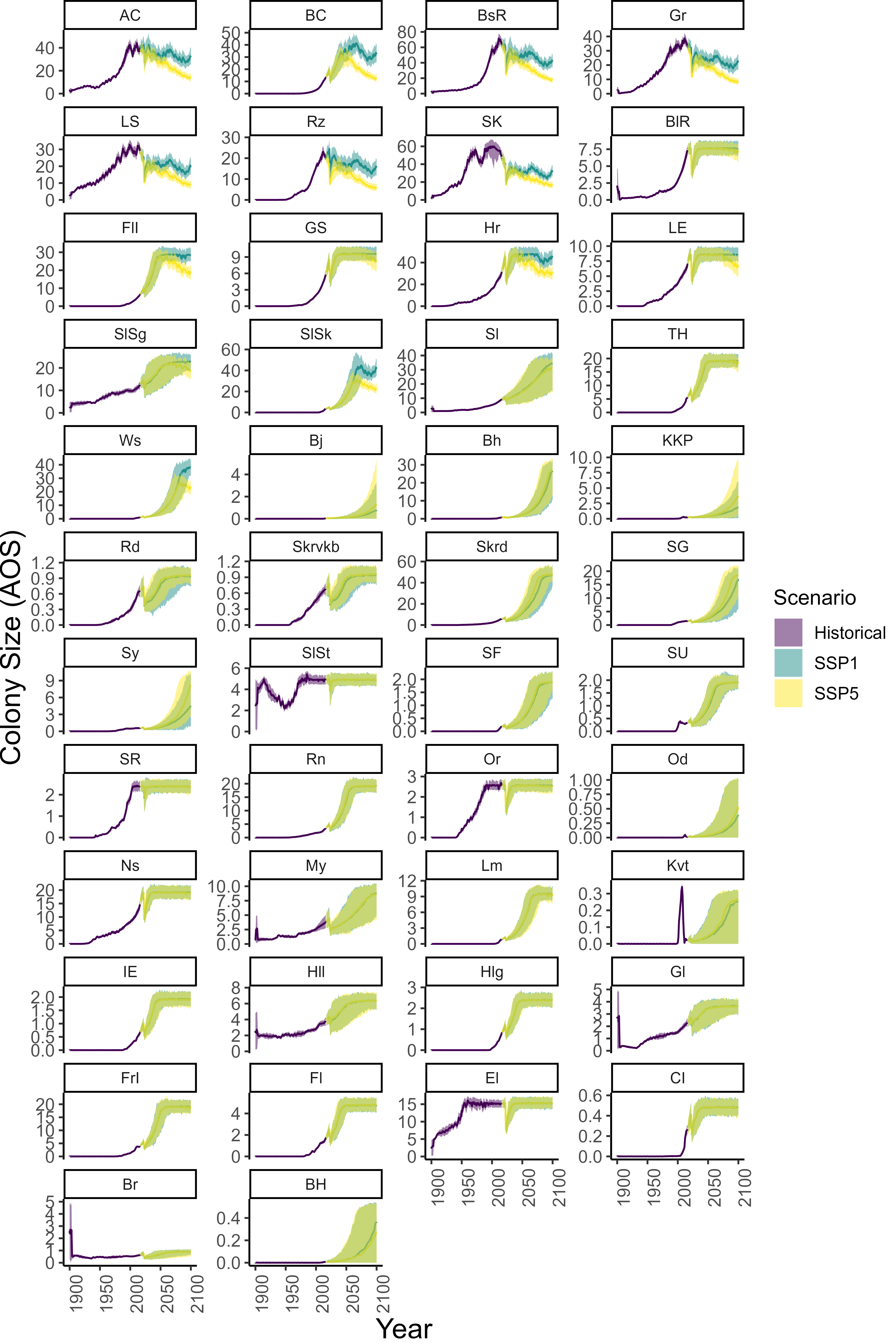

Supplement: Supplementary file 6 — Figure S6: Posterior estimates of median colony sizes and credible interval spanning the years 1900–2016 (violet) and future colony size predictions under climate change scenario SSP1 (green) and under SSP5 (yellow) for all extant gannet colonies of the Northeast Atlantic metapopulation. [file ELE-27-0-s003.tiff]

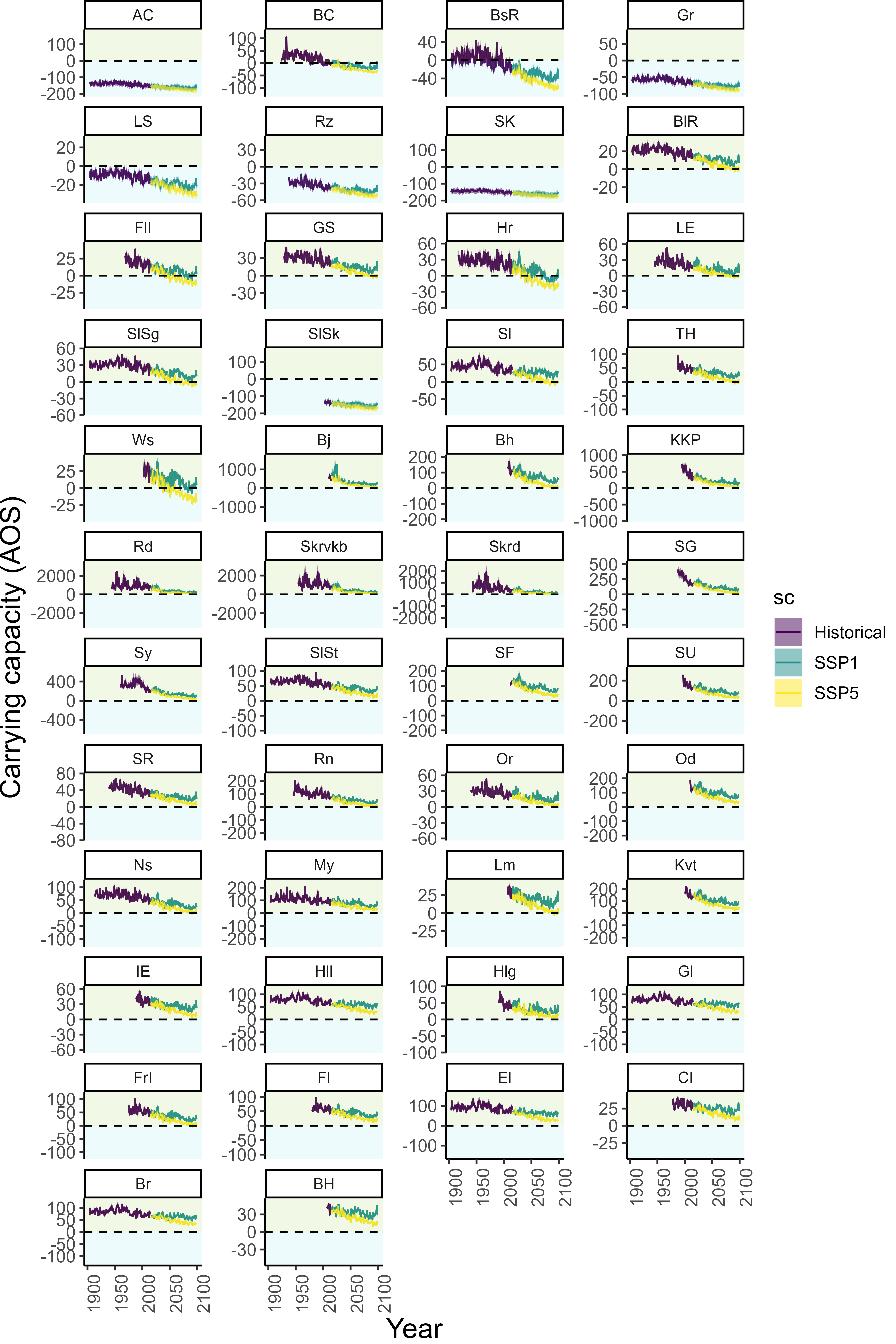

Supplement: Supplementary file 7 — Figure S7: Posterior estimates of colony carrying capacities spanning the years 1900–2016 (violet) and future predictions under climate change scenario SSP1 (green) and under SSP5 (yellow) for all extant gannet colonies of the Northeast Atlantic metapopulation. Background colours in green pertain to terrestrial regulation, areas in blue to marine regulation. [file ELE-27-0-s007.tiff]
