# Supplementary material for: Past and future effects of climate on the metapopulation dynamics of a Northeast Atlantic seabird across two centuries
Source: Ecol Lett. 2024 Dec 31;27(12):e14479. doi: 10.1111/ele.14479 (PMC11686948; doi:10.1111/ele.14479)
Supplement: Supplementary file 8 — Table S1: Model ID and institution ID of model used for covariate data. [file ELE-27-0-s002.docx]

Supporting information

Past and future effects of climate on the metapopulation dynamics of a NorthEast Atlantic seabird across two centuries

Jana W. E. Jeglinski, Holly I. Niven, Sarah Wanless, Robert T. Barrett, Mike P. Harris, Jochen Dierschke and Jason Matthiopoulos

Table 1: Model ID and institution ID of model used for covariate data.

| Model ID | Institution | Institution ID |
| --- | --- | --- |
| HadGEM3-GC31-MM | Met Office Hadley Centre | MOHC |
